# Evolving strategies in obstructive hypertrophic cardiomyopathy: myosin inhibitors as monotherapy compared with beta-blockers

**DOI:** 10.1007/s10741-026-10603-9

**Published:** 2026-03-18

**Authors:** Avishay Grupper, Aaron L. Sverdlov, Baljash S. Cheema, Amir Nasrollahizadeh, Kaveh Hosseini

**Affiliations:** 1 Shamir Medical Center, Zerifin, Be’er Ya’akov, Israel; 2 Gray Faculty of Medical and Health Sciences, Tel Aviv University, Tel Aviv-Yafo, Israel; 3 Cardiovascular Department, John Hunter Hospital, New Lambton Heights, NSW Australia; 4 School of Medicine and Public Health, University of Newcastle, Callaghan, NSW Australia; 5 Division of Cardiology, Department of Medicine, Northwestern University Feinberg School of Medicine, Chicago, IL USA; 6 Cardiovascular Diseases Research Institute, Tehran University of Medical Sciences, Tehran, Iran; 7 Department of Cardiology, Copenhagen University Hospital - Herlev and Gentofte, Copenhagen, Denmark; 8 Center for Translational Cardiology and Pragmatic Randomized Trials, Department of Biomedical Sciences, Faculty of Health and Medical Sciences, University of Copenhagen, Copenhagen, Denmark

**Keywords:** Obstructive hypertrophic cardiomyopathy, Cardiac myosin inhibitors, Mavacamten, Aficamten, Beta-blockers, Left ventricular outflow tract obstruction, Sarcomere, Pharmacologic therapy

## Abstract

Obstructive hypertrophic cardiomyopathy (oHCM) is characterized by asymmetric hypertrophy, left ventricular outflow tract (LVOT) obstruction, and impaired diastolic function. β-adrenergic blockers (BB) have long been the cornerstone of medical therapy, providing symptomatic improvement but without disease-modifying effects. Cardiac myosin inhibitors (CMIs), including mavacamten and aficamten, represent a new therapeutic class targeting the hypercontractile sarcomere. Randomized trials have demonstrated substantial reductions in LVOT gradients and improvements in exercise capacity, New York Heart Association functional class, and quality of life, with an acceptable safety profile. Subgroup analyses from EXPLORER-HCM and FOREST-HCM suggested that concomitant BB therapy may blunt the exercise capacity benefits of CMIs. The MAPLE-HCM trial provided the first direct head-to-head comparison, showing that aficamten monotherapy was superior to metoprolol in improving peak oxygen uptake, functional status, and hemodynamic parameters. Current evidence indicates that CMIs offer greater efficacy than BB in improving exercise tolerance and LVOT obstruction in oHCM, supporting their role as potential first-line therapy. However, long-term safety, durability of benefit, optimal sequencing, and patient selection require further study. BBs remain clinically relevant for specific patient subgroups, but the advent of CMIs marks a paradigm shift from symptomatic relief toward targeted disease modification in oHCM.

**Graphical abstract:**

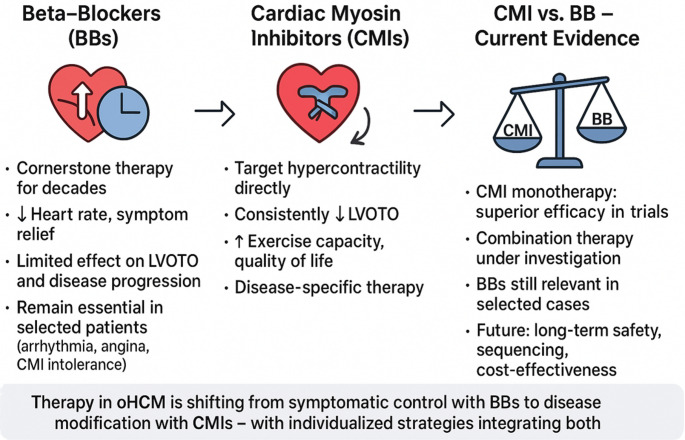

## Introduction

Hypertrophic cardiomyopathy (HCM) is the most common genetic cardiomyopathy, characterized by asymmetric left ventricular hypertrophy. HCM encompasses both obstructive and non-obstructive phenotypes, with non-obstructive HCM often characterized by prominent diastolic dysfunction that may be a major driver of symptoms. Obstructive hypertrophic cardiomyopathy (oHCM), a distinct subset of HCM, is characterized by asymmetric left ventricular hypertrophy, dynamic left ventricular outflow tract (LVOT) obstruction, and impaired diastolic function. Patients frequently present with exertional dyspnea, angina, palpitations, syncope, or heart failure symptoms, and the disease remains an important cause of sudden cardiac death in young adults [1–3].

Pharmacological therapy has long relied on negative inotropic agents, with β-adrenergic blockers (BB) established as the first-line treatment for symptomatic patients [2, 3]. Through their negative inotropic effects, BB mitigate left ventricular contractility and dynamic LVOT gradients, leading to improved symptoms in many patients. Their role has been supported by decades of clinical use, despite lack of robust randomized trial evidence [4].

A major shift in the therapeutic paradigm has occurred with the development of cardiac myosin inhibitors (CMI). These agents directly target the underlying pathophysiology of hypertrophic cardiomyopathy (HCM) by reducing excessive actin–myosin cross-bridge formation and left ventricular hypercontractility [5]. Preclinical studies demonstrate that CMIs stabilize myosin in a super-relaxed state, reduce myosin ATPase activity, and decrease the number of force-generating cross-bridges, thereby improving myocardial energetic efficiency and facilitating diastolic relaxation—mechanistic effects that translate into symptom relief in obstructive HCM via reduction of dynamic LVOT obstruction [5]. Mavacamten and aficamten, oral CMIs, have demonstrated marked reductions in LVOT gradient and improvements in functional capacity and quality of life in early clinical studies and pivotal randomized trials [6–9].

The advantages of myosin inhibition have prompted re-evaluation regarding the continued role of BB in oHCM management. Combination therapy with BB and CMI has been explored in clinical practice, but recent trial data suggest that myosin inhibitors may be effective as monotherapy and could potentially replace traditional negative inotropes in selected patients [10].

This review will provide a historical overview of BB therapy in oHCM, summarize available data on combination strategies with myosin inhibitors, and examine emerging evidence on myosin inhibitor monotherapy compared with BB, with a focus on the evolving therapeutic paradigm.

## Beta-blockers in the treatment of oHCM

Non-vasodilating BB, such as metoprolol, sotalol, and propranolol, have long represented the cornerstone of pharmacological management for symptomatic oHCM and remain first-line monotherapy according to recent international guidelines [2, 3]. Their therapeutic benefit derives primarily from negative inotropic effects that reduce myocardial contractility and attenuate dynamic LVOT obstruction [11]. While BB also reduce heart rate and myocardial oxygen demand, their symptomatic benefit in oHCM is largely attributable to the reduction in LVOT gradients rather than improvement in diastolic filling [12]. Moreover, BB help attenuate the dynamic LVOT obstruction, which is known to vary with autonomic tone, posture, and physical activity [13]. Unlike vasodilating agents, non-vasodilating BB avoids afterload reduction, thereby preventing exacerbation of the LVOT gradient and obstructive symptoms [14].

The clinical evidence supporting BB in oHCM has historically been derived from early observational studies and small placebo-controlled trials demonstrating symptomatic improvement. More recently, the TEMPO RCT of 29 participants showed that metoprolol (typically at 150 mg daily) significantly reduced LVOT gradients at rest and peak exercise and improved NYHA class, CCS angina class, and KCCQ overall summary score, but did not improve peak exercise oxygen consumption or exercise capacity versus placebo [15]. In the TEMPO invasive exercise-hemodynamic sub study (*n* = 28), metoprolol did not change PCWP at rest or peak exercise (nor ΔPCWP), despite lowering heart rate and LVOT gradient, providing mechanistic context for why symptom/QoL gains may not translate into improved maximal exercise performance [16].

Treatment strategies typically emphasize titration to the maximally tolerated dose, with the primary goal of symptom relief rather than LVOT gradient reduction alone, aiming to improve patient-reported outcomes [15–17]. In patients with comorbidities such as hypertension or atrial fibrillation requiring rate control, BB are particularly advantageous, addressing both conditions simultaneously [18, 19]. Furthermore, in pregnant women with oHCM, selected agents such as metoprolol are considered acceptable for symptom management when accompanied by fetal monitoring [20].

Although BBs have maintained a central role in the management of oHCM, they do not modify the underlying disease process, as they neither reverse hypertrophy nor prevent disease progression [21]. In clinical practice, many patients continue to experience persistent symptoms that necessitate the addition of second-line pharmacological agents or surgical interventions [2, 3]. The partial therapeutic effect, intolerance in some patients, and reliance on older, limited evidence highlights the therapeutic limitations of BB [22] and underscore the importance of contemporary head-to-head comparisons, such as the MAPLE-HCM trial, evaluating BB against novel agents like CMI [10].

## Cardiac myosin inhibitors in the treatment of oHCM

### Preclinical evidence

CMIs represent the first therapeutic class developed to directly target the pathophysiology of HCM, a disease caused by genetic variants in multiple sarcomeric genes [23]. These variants have various influences on transcriptional efficiency and protein function, due to incomplete penetrance, variable expressivity, and phenotypic heterogeneity. Nevertheless, hypercontractility and excessive force generation are considered key unifying mechanisms underlying the clinical manifestations of HCM [24]. Unlike conventional therapies such as BB or calcium channel blockers, which are primarily directed at symptom relief, CMIs reduce sarcomere power output by stabilizing myosin heads in an energetically favorable state, thereby attenuating hypercontractility [23]. In preclinical animal studies, mavacamten, the first-in-class CMI, was shown to slow the development of left ventricular hypertrophy, reduce fibrosis, and restore the typical myocyte disarray to a more organized myocardial architecture [5].

### Clinical trials with mavacamten

The pivotal EXPLORER-HCM trial demonstrated that mavacamten significantly improved exercise capacity, measured by peak oxygen uptake (pVO₂), NYHA functional class, LVOT gradient, biomarkers of cardiac stress, and quality of life compared with placebo [7]. These benefits established proof-of-concept that targeted sarcomeric modulation can translate into meaningful functional improvement in symptomatic obstructive HCM. A minority of patients experienced transient reductions in left ventricular ejection fraction (LVEF); however, these changes were reversible with dose adjustment or treatment discontinuation, underscoring the importance of structured echocardiographic surveillance and individualized titration [7].

Longer-term follow-up from EXPLORER-HCM and its extension studies confirmed the durability of symptomatic and hemodynamic benefit, while also highlighting the need for careful monitoring in the context of mavacamten’s long half-life and narrow therapeutic window. Importantly, these trials helped define contemporary clinical workflows for CMI use, including echocardiography-guided dosing algorithms and risk mitigation strategies [25].

In the VALOR-HCM trial, mavacamten significantly reduced the need for septal reduction therapy among patients with advanced symptoms and severe resting or provoked LVOT obstruction, while improving functional status, hemodynamics, and biomarkers [8]. Beyond symptomatic relief, these findings suggested that sustained reduction in hypercontractility may alter the natural history of obstruction in selected patients, supporting the concept of CMIs as potential disease-modifying agents rather than purely symptomatic therapies.

### Clinical trials with aficamten

Aficamten, a second-generation cardiac myosin inhibitor, was designed to address several pharmacokinetic and practical limitations observed with mavacamten, including its prolonged half-life, dependence on cytochrome P450 metabolism, and narrow therapeutic margin [26]. Its shorter half-life, reduced potential for drug–drug interactions, and wider therapeutic window allow for more rapid titration and potentially improved safety management, particularly in patients requiring dynamic dose adjustments [26].

Randomized and extension studies have consistently demonstrated that aficamten improves exercise capacity, symptoms, LVOT gradients, and cardiac biomarkers in patients with obstructive HCM. In the SEQUOIA-HCM trial, aficamten led to significant increases in pVO₂, improvements in NYHA functional class and quality-of-life measures and marked reductions in LVOT gradients [9]. Notably, reductions in LVEF were generally modest, predictable, and reversible, supporting the hypothesis that pharmacokinetic properties may translate into more flexible and potentially safer clinical use.

Collectively, aficamten trial data reflect an evolution in CMI development, emphasizing pharmacologic precision, titration flexibility, and real-world applicability. These features are particularly relevant in the context of emerging studies such as MAPLE-HCM, which aim to refine patient selection and optimize therapeutic strategies across the obstructive HCM spectrum.

### Concomitant use of CMIs with BB

At the time these trials were conducted, BB remained the standard first-line therapy for symptomatic oHCM. Accordingly, most patients enrolled in the landmark CMIs clinical studies received background BB therapy. In EXPLORER-HCM, 76% of patients in the mavacamten arm and 74% in the placebo arm were treated with BB at baseline [7], with comparable rates reported in VALOR-HCM [8]. In the SEQUOIA-HCM trial, 61% of participants were already receiving BB at baseline (61% in the aficamten arm and 62% the placebo arm) [9].

These trials collectively suggest that MCIs have robust efficacy in oHCM, both as add-on therapy and with potential to function as a monotherapy in selected patients, although head-to-head clinical trials directly comparing long-term outcomes in CMIs against BB were lacking.

## Cardiac myosin inhibitors versus beta-blockers in oHCM

In a prespecified analysis of the EXPLORER-HCM trial, the magnitude of treatment benefit with mavacamten differed according to baseline BB use. Patients not receiving BBs demonstrated a substantially greater improvement in the composite primary endpoint compared with those on BBs (between-group difference: 52.6% vs. 8.7%) [27]. The BB subgroup exhibited lower baseline and on-treatment peak exercise heart rates (119 vs. 138 bpm) and achieved a smaller increase in pVO₂ at week 30 (+ 1.1 vs. +2.2 mL/kg/min) [27]. By contrast, heart rate independent endpoints, including VE/VCO₂ slope, LVOT gradient, and NYHA class, improved with mavacamten regardless of BB use [27]. These findings may suggest that BBs attenuate heart rate response due to chronotropic limitations, However, another potential explanation to the discrepancy results are different in disease severity between the 2 groups, with more significant symptoms at baseline in the BB group.

In the FOREST-HCM open-label extension trial with aficamten, many patients receiving background therapies, including BBs, were able to reduce or discontinue these medications without loss of efficacy. Improvements in symptoms, hemodynamics (LVOT gradients), and biomarkers were maintained in patients who minimized or discontinued background therapies [28]. While these extension data indicate many patients can reduce or discontinue background negative inotropes without loss of CMI benefit, abrupt withdrawal of chronic BB in HCM can provoke transient adrenergic hypersensitivity with adverse clinical consequences [29]. Thus, stepwise down-titration, rather than abrupt cessation, of background negative inotropes during CMI initiation is prudent, with close monitoring and re-introduction if symptoms recur. In interpreting head-to-head comparisons, it is notable that TEMPO showed metoprolol can improve obstruction-linked surrogates and health status without improving peak VO₂ versus placebo, supporting the concept that chronotropic limitation may blunt objective exercise capacity gains even when symptoms improve [15, 16].

MAPLE-HCM represents the first randomized, double-blind, double-dummy head-to-head trial directly comparing a CMI, aficamten, with the BB metoprolol as monotherapy [10]. The primary endpoint was change in pVO₂ at 24 weeks. A total of 175 adults with symptomatic oHCM were randomized to aficamten (5–20 mg) or metoprolol (50–200 mg). Importantly, 76% underwent pre-randomization washout of prior therapy, ensuring a true monotherapy comparison. Aficamten significantly improved pVO₂ (+ 1.1 mL/kg/min) compared with a decline in the metoprolol group (− 1.2 mL/kg/min), yielding a least-squares difference of + 2.3 mL/kg/min (*P* < 0.001) [10]. The decline in peak VO₂ with metoprolol monotherapy in MAPLE-HCM is consistent with the possibility that heart-rate suppression without meaningful obstruction relief can worsen objective exercise capacity. Indeed, the trial reports a clear on-target chronotropic effect, while improvements in LVOT gradient and key objective markers were not seen with metoprolol despite subjective improvements in NYHA class and KCCQ-CSS. Key secondary endpoints all favored aficamten, including greater NYHA class improvement, higher quality of life assessments by KCCQ-CSS scores, and larger reductions in post-Valsalva LVOT gradient, NT-pro BNP, and left atrial volume index [10]. LV mass index did not differ significantly between groups, and adverse event rates were similar, apart from a modest, mechanism-consistent reduction in LVEF (~ 4% absolute) in the aficamten arm [10].

An echocardiographic substudy of MAPLE-HCM provided further mechanistic insights: aficamten produced larger reductions in LVOT gradients, decreased left atrial volume index, improved diastolic function, and reduced systolic anterior motion and mitral regurgitation, while resting cardiac output remained unchanged relative to metoprolol [30].

While these findings are strongly favorable, certain limitations should be acknowledged, including the relatively short 24-week trial duration and questions regarding generalizability to BBs other than metoprolol. Nevertheless, the interaction signal observed in EXPLORER and FOREST trails together with MAPLE’s direct head-to-head comparison support the consideration of a CMI-first monotherapy approach, particularly when improving exercise capacity and hemodynamic parameters is the therapeutic priority in patients with oHCM.

## Summary

Despite marked advances in oHCM treatment, several limitations remain. Current data on head-to-head comparisons between CMIs and BB are limited, with most evidence derived from subgroup analyses or a single randomized trial. Long-term safety, durability of response, and optimal patient selection for CMI monotherapy versus combination therapy are not fully defined. Moreover, questions regarding treatment sequencing, cost-effectiveness, and real-world applicability require further clarification.

Importantly, the current data presented in this review does not eliminate the clinical relevance of BBs but rather highlights the need for individualized therapy tailored to patient profile and treatment goals. BBs remain an essential option in selected patients, particularly those with concomitant arrhythmias, angina, or intolerance to newer therapies.

Looking forward, ongoing and future studies will determine whether CMIs should be established as first-line therapy in oHCM and define their role relative to conventional agents. Integration of CMIs into clinical practice marks a paradigm shift from symptomatic control to targeted disease modification, but careful evaluation of long-term outcomes and individualized treatment strategies will be essential.

## Data Availability

No datasets were generated or analysed during the current study.
